# Pharyngomalacia in Neonates: The Missed Issue

**DOI:** 10.3389/fped.2020.555564

**Published:** 2020-10-30

**Authors:** Mohammad Ashkan Moslehi

**Affiliations:** Pediatric Interventional Pulmonology Division, Department of Pediatrics, School of Medicine, Shiraz University of Medical Sciences, Shiraz, Iran

**Keywords:** neonates, stridor, pharyngomalacia, airway malacia, noisy breathing

## Abstract

**Background:** Airway malacia (AM) is a weakness of the airway's frameworks making them collapsible during the respiratory phases. Although the larynx, trachea, and bronchus are the usual sites for malacia to occur, there is another important type of malacia that involves the pharynx. Pharyngomalacia (PM) or concentric pharyngeal wall inspiratory collapse (PWIC) is mostly missed during bronchoscopic evaluations in the neonates with noisy breathing because people are not aware of this condition.

**Methods:** This study aimed to evaluate the nasopharyngeal investigation among neonates suffering from noisy breathing. The retrospective study was undertaken to assess the frequency of PM and to propose indications for intervention in 100 neonates with noisy breathing. A thin fiberoptic bronchoscope was used to evaluate the upper airways under conscious status without any sedation in the neonates.

**Results:** A total of 100 neonates with noisy breathing from September 2015 to October 2018 were retrospectively analyzed. The most common presenting symptom was inspiratory stridor which was observed in 35 (92.1%) of cases. PM was diagnosed in 38 neonates (38%) including 27 (71%) males and 13 (29%) females. Seventeen (44.7%) cases had mild, 11 (28.9%) cases had moderate, and 10 (26.4%) cases had a severe type of PM. PM was more prominent at the velopharynx level in 15 (39.4%) cases, and it was accompanied by up to six synchronous airway abnormalities. The most frequent synchronous airway abnormality was laryngomalacia in 13 (34.3%).

**Conclusion:** PM is one of the causes of noisy breathing in infants. Since PM can be accompanied by the presence of other types of airway malacia, the issue becomes more complicated. On the other hand, lack of experience and facilities are two main causes for the accurate diagnosis and effective management among neonates. This study indicates that the investigation of pharynx is a missed part of the many workups that are used to diagnose the site of involvement in neonates with noisy breathing.

## Introduction

The upper airway is defined anatomically as the airway segment between the nostrils and trachea at the level of the thoracic inlet, including five compartments: the nasopharynx (functional during nasopharyngeal breathing), oropharynx cavity (functional during oropharyngeal breathing), the hypopharynx, the larynx, and upper third part of the trachea within the level of the thoracic inlet. Due to the parallel anatomic arrangement between the oral and nasal cavities, they are rarely the site of upper airway obstruction, except in cases of congenital malformation, massive head, and neck trauma and injuries from burns. Considering the pathophysiology of upper airway Obstruction (UAO), it is divided into dynamic (variable) and anatomical (fixed) obstruction. The major symptoms of UAO in neonates include noisy breathing and dyspnea. The severity of the symptoms directly depends on the severity of the obstruction. Noisy breathing in neonates is defined as unusual respiratory sounds during respiratory phases. The most important unusual sounds in neonates are stridor, wheezing, and snoring. Inspiratory stridor is the main symptom resulting from extra-thoracic airway partial obstruction. Differential diagnosis of stridor can be divided based on anatomical levels including supralaryngeal, laryngeal, and tracheal categories. Supralaryngeal causes of stridor consist of supralaryngeal causes which include vallecular cysts, thyroglossal cysts, and tongue dermoid or teratoma (1). Isolated pharyngomalacia is considered one of the causes in selected neonates with severe stridor (2). When the obstruction is acute and complete, such as bilateral choanal atresia, sudden dyspnea, or even suffocation may result at birth. But in chronic onset and uncompleted UAO, the neonate develops noisy breathing and dyspnea over time, especially during increased respiratory works such as feeding, crying, or sleeping in a prone position. The symptoms of dyspnea and noisy breathing are identical to those experienced with other types of airway malacia, such as tracheomalacia, bronchomalacia, or both. These similarities can lead to diagnostic confusion. Moreover, not all bronchoscopies looked at the pharynx for more investigation, and this makes pharyngomalacia a rarely diagnosed condition. This study was performed since the impact of PM as the major type of the UAO and its collaboration with other airway obstructions has not been well-understood yet.

## Materials and Methods

This retrospective study was done to investigate the role and the frequency of PM as a cause of noisy breathing in 100 neonates for 3 years. All records of neonates referred to our center with noisy breathing from September 2015 to October 2018 were included for reviewing. Bronchoscopy investigation is a routine investigation technique in neonates with noisy breathing that includes the following criteria: age <28 days, noisy breathing, dyspnea, cyanotic cough spells, aspiration and cyanotic spells during feeding, and recurrent apnea episodes. Aspiration is clinically defined as presenting choking, cyanosis, and apnea during feeding and is confirmed through fiberoptic endoscopic evaluation of swallowing (FEES) and barium swallow study (BSS). Exclusion criteria are as follows: age more than 1 month, parental disagreements, and no nasopharyngeal endoscopy examination.

The study was approved by the hospital ethical committee, and the parents or caregiver gave their informed consent to be enrolled in the study. Moreover, written informed consent was obtained from all the parents for both the publication and any accompanying images.

The assessment of upper airway dynamics was emphasized by using a thin (2.8 mm) flexible bronchoscope (Olympus Company, Japan) when the patients were on their spontaneous breathing with a nasal approach. Since different levels of sedation led to different dynamic and functional results and there was the possibility of interference of the results in the bronchoscopy evaluations, neither generalized anesthesia nor sedation was administered. For minimizing the possibility of airway irritation and spasm, lidocaine gel (2%) was applied all over the exterior surface of the FFB flexible bronchoscope. Based on the established protocol at the authors' department, a nasal cannula was inserted through one of the nostrils down to the nasopharynx to maintain oxygenation. No PEEP or positive ventilation was used at the time of endoscopy evaluations as it could interfere with the flaccidity of airway walls and the final reports. According to the bronchoscopy investigation, PM was defined in multiple sites as follows: from the level of the hard palate to the tip of the uvula (velopharynx), from the end of the uvula to the tip of the epiglottis (oropharynx), and finally from the tip of the epiglottis to the vocal cords (hypopharynx). As to the best of the author's knowledge, there were not any grading scales regarding the severity of obstruction in PM. Thus, the author used a new modified grading scale based on the one that has been used for adenoid hyperplasia and tracheomalacia depending on the present airway obstruction and severity of the symptoms. So, it has been graded as mild, moderate, and severe when an obstruction was <50%, between 50 and 75%, and more than 75% of the airway diameter, respectively.

To prove the exact role of PM in induced symptoms, positive pressure ventilation of 5 cm water was applied by closing the anesthesia bag valve and bypassing the obstruction level through using the nasopharyngeal tube. These maneuvers aborted the pharyngeal wall collapse and the symptoms. Patients affected by mild to severe PM benefited from supportive medical management while airway structural stability was expected with increasing age. The supportive care used included nasal washing with normal saline, nasopharyngeal mucosal gentle suctioning, antireflux positions, and therapy with oral proton-pump inhibitors (PPIs) like omeprazole, nasopharyngeal catheters, continuous positive airway pressure (CPAP), or bilevel positive airway pressure (BiPAP).

All patients underwent a follow-up program adjusted to individual patients' conditions and needs in terms of the different types of PM every 2–3 months for up to 3 years.

## Results

**Figure 1:**
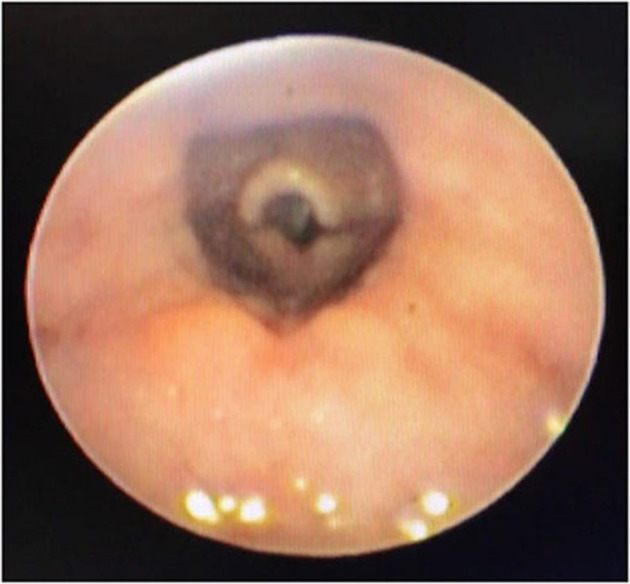
Moderate PM.

**Figure 2:**
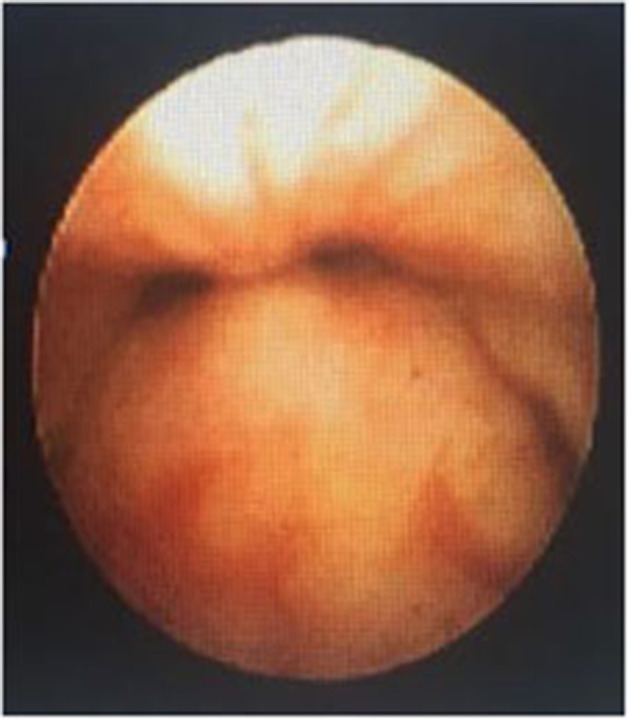
Severe PM.

In a retrospective review of consecutive endoscopic evaluations and clinical data of 100 (48 female and 52 male) neonates with UAO, PM was diagnosed in 38 neonates (38%) including 25(65.8%) males and 13 (34.2%) females. The age of neonates at the time of endoscopy diagnosis was from 7 to 28 days after birth. With regard to the extent of PM, the number (present) of children with mild, moderate (Figure 1), and severe (Figure 2) were 17 (44.7%), 11 (28.9%), and 10 (26.4%), respectively. Based on the results of the bronchoscopy investigations, PM was most prominent at the velopharynx level 15 (39.4%) followed by hypopharynx 13 (34.2%), the whole length of pharynx 7 (18.4%), and oropharynx 3 (8%). The most common presenting symptoms were inspiratory stridor 35 (92.1%), cyanotic cough spell 27 (71%), aspiration and cyanotic spells during feeding 21 (55.2%), and recurrent apnea 18 (47.3%).

This study also showed that PM was accompanied by up to six synchronous airway abnormalities; the most frequent ones were laryngomalacia 13 (34.3%), tracheomalacia 12 (31.5%), bronchomalacia 6 (15.8%), larygotracheobronchomalacia 3 (8%), tracheoesophageal fistula (TEF) 2 (5.2%), and posterior laryngeal cleft 2 (5.2%). The results are presented in Table 1. Generalized hypotonia was the most common associated systemic finding 4 (12%). There were two neonates with Down syndrome and one with VACTER syndrome suffering from severe PM at the level of velopharynx.

**Table 1:** Detailed data.

**Demographics**	***n* = 38**
Sex (male)	25 (65.8%)^*^
Sex (female)	13 (34.2%)^*^
Birth gestational age (weeks)	36 (25-41)^+^
Age at the time of bronchoscopy (days)	16 (7-28)^+^
**Severity**	
Mild	17 (44.7%)^*^
Moderate	11 (28.9%)^*^
Severe	10 (26.4%)^*^
**Site of maximum involvement**	
Velopharynx	15 (39.4%)^*^
Hypopharynx	13 (34.2%)^*^
Whole length of pharynx	7 (18.4%)^*^
Oropharynx	3 (8%)^*^
**Symptoms**	
Stridor	35 (92.1%)^*^
Cyanotic cough spell	27 (71%)^*^
Aspiration (cyanotic spells during feeding)	21 (55.2%)^*^
Recurrent apnea	18 (47.3%)^*^
**Synchronous airway abnormalities**	
Laryngomalacia	13 (34.3%)^*^
Tracheomalacia	12 (31.5%)^*^
Bronchomalacia	6 (15.8%)^*^
Larygotracheobronchomalacia	3 (8%)^*^
Tracheoesophageal fistula (TEF)	2 (5.2%)^*^
Posterior laryngeal cleft	2 (5.2%)^*^

* *Frequency (percentage)*.

+ *Median (min-max)*.

Non-invasive positive ventilation devices including CPAP and BiPAP were used in 21 (55.3%) neonates with moderate to severe PM. The duration of use of these devices varied depending on the age and severity of the PM (mean, 7 ± 3.5 months). Four (10.5%) cases including one patient with VACTER, one with Down syndrome, one with posterior laryngeal cleft, and one with TEF underwent tracheostomy.

In the follow-up, 35 (92%) of the cases became asymptomatic from 10 to 24 months (mean, 18 ± 5 months) by using supportive care.

## Discussion

Noisy breathing is one of the most common indications for doing bronchoscopic investigations in neonates. UAO and LAO are the main causes of these abnormal breathing sounds. UAO is a potentially severe and life-threatening complication among neonates. Tracheomalacia is the most common congenital UAO among this age group (1). Its incidence seems to be underestimated but reported as about 1 in every 2,100 children (3). Most children are either asymptomatic or minimally symptomatic, and most cases involve posterior malacia of the trachea, with associated broad tracheal rings. On the other hand, PM is a dynamic obstruction of the air column proximal to the glottis during inspiration. The author's objective was to assess PM's incidence and its contribution to the symptoms of UAO and to propose indications for intervention.

As 38% of neonates had various types of pharyngomalacia, this study showed that there are some important misdiagnoses in previous studies regarding the causes of noisy breathing in neonates. A wide variety of PM exists that is often missed first, as the oral approach is the often-used routine bronchoscopy route for any investigations done by a pulmonologist. Secondly, most pediatric bronchoscopists may not be familiar with the anatomical and physiological aspects of the upper airways especially in the neonates and young infants. Thirdly, it is difficult to do the bronchoscopy setup in small neonates especially in the unstable ones, and lastly, neonatal bronchoscopy facilities are unavailable in many centers. Based on this study, male neonates were more prone to have PM than females. There is a scant research regarding the related issue in the literature. However, based on bronchoscopic investigations, this may be because male neonates have longer velopharynx compared with female neonates (4). Previously, laryngomalacia (87.2%), pharyngomalacia (33.3%), and tracheomalacia (10.3%) were reported as the three most prevalent findings on endoscopy of neonates with sleep apnea (5). PM was also reported as an under-recognized rare condition in patients with Down syndrome who had sleep apnea (6). Thus, PM may account for one of the major causes of obstructive sleep apnea. In the study by Shatz et al., the majority of children had obstruction related to pharyngeal hypotonia and collapse. They also mentioned that PM can lead to prolonged hospitalization and intensive care admission and may raise the difficulty in management issues (7). The severity of PM was measured through severe retractions and respiratory compromises like respiratory failure, apnea, cyanotic spells, the need for more respiratory supports depending on additional oxygen, extubation failure, choking spells while feeding, and poor weight gain. Moreover, as there were no standard severity grading scales especially in neonates, the author considers the percentage of stenosis as the described severity scales from mild to severe. According to this scaling, most of the cases had mild to moderate PM.

Similar to other types of airway malacia, PM seems to be a self-limiting condition and spontaneous recovery occurred within 36 months. Although mild PM is watched expectantly and anticipated to improve with time, there were more severe symptomatic PMs.When it occurs with other airway insults, it warrants interventions like non-invasive ventilatory supports (nasopharyngeal catheters, CPAP, bilevel instruments) and rarely may need surgery in some cases (8, 9).

In this study, most of the index cases (92%) became asymptomatic due to supportive care including antireflux position and therapy, the decrease of mucosal stickiness of the nasopharyngeal wall with nasal spray 10–15 min before feeding and 3–5 ml normal saline oral solution after feeding, gentle nasal suctioning, and small frequent feeding.

In neonates with compromised airway disorders where swallowing interrupts with normal smooth breathing, this causes sucking-swallowing discoordination (SSD) leading to aspiration although the exact incidence is not well-established (10, 11).

In this study, more than half of the patients had aspiration due to SSD clinically presented with cyanotic spells during feeding and was approved by FEES and BSS. Direct vision investigation with FEES study revealed that aspiration became effectively diminished by resolving the PM-induced partial obstruction using nasopharyngeal bypassing, gentle mucosal suctioning, and patient positioning with 30–45% of body (not neck flection) elevation.

Progressive and severe PM cases required a nasopharyngeal catheter for bypassing the stenotic level; CPAP or BiPAP form weeks to months. Four cases underwent tracheostomy procedures especially those with concomitant syndromes (VACTERL and Down syndromes). It seems the rest of the cases became asymptomatic as they were followed up until the time of publishing the data.

This study has some limitations including the nature of retrospective studies, lack of previous research studies on the topic, limited access to data, and a combination of abnormalities which on their own can cause noisy breathing.

The incidence of PM among neonates with UAO was beyond expectations in this study, and its role in UAO deserves greater recognition. This study also showed a combination of abnormalities, which on their own can cause noisy breathing. Its commonly associated abnormalities can include laryngeal clefts, TEF, and bronchomalacia. The current gold standard for the diagnosis of PM is a dynamic evaluation under direct vision through nasopharyngoscopy. A better diagnosis of PM will improve the treatment of UAO.

## Data Availability Statement

The original contributions presented in the study are included in the article/supplementary materials, further inquiries can be directed to the corresponding author/s.

## Ethics Statement

Ethical review and approval was not required for the study on human participants in accordance with the local legislation and institutional requirements. Written informed consent to participate in this study was provided by the participants' legal guardian/next of kin.

## Author Contributions

MM wrote the manuscript and approved it for publication.

## Conflict of Interest

The author declares that the research was conducted in the absence of any commercial or financial relationships that could be construed as a potential conflict of interest.
